# 1-(2-Bromo­ethyl)-1,4-diazo­niabicyclo­[2.2.2]octane bromide tetra­fluoro­borate

**DOI:** 10.1107/S1600536810017204

**Published:** 2010-05-15

**Authors:** Jing-mei Xiao

**Affiliations:** a Ordered Matter Science Research Center, College of Chemistry and Chemical Engineering, Southeast University, Nanjing 211189, People’s Republic of China

## Abstract

In the crystal of the title compound, C_8_H_17_BrN_2_^2+^·Br^−^·BF_4_^−^, a weak inter­molecular N—H⋯Br hydrogen bond is observed between the cation and the bromide anion. A two-part disorder model was applied to the BF_4_^−^ anion with a refined major–minor occupancy ratio of 0.837 (14):0.163 (14).

## Related literature

For the crystal structures and properties of related compounds, see: Chen *et al.*(2009 ▶); Fu *et al.* (2009 ▶); Zhao *et al.* (2008 ▶).
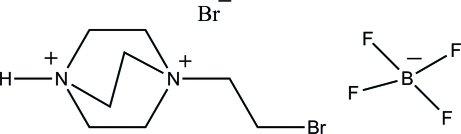


## Experimental

### 

#### Crystal data

C_8_H_17_BrN_2_^2+^·BF_4_^−^·Br^−^
*M* *_r_* = 387.87
Orthorhombic, 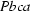

*a* = 11.972 (2) Å
*b* = 10.782 (2) Å
*c* = 21.165 (4) Å
*V* = 2732.0 (10) Å^3^
*Z* = 8
Mo *K*α radiation
μ = 5.96 mm^−1^
*T* = 293 K
0.3 × 0.25 × 0.2 mm

#### Data collection

Rigaku Mercury2 diffractometer
Absorption correction: multi-scan (*CrystalClear*; Rigaku, 2005 ▶) *T*_min_ = 0.191, *T*_max_ = 0.303
26313 measured reflections
3124 independent reflections
1881 reflections with *I* > 2σ(*I*)
*R*_int_ = 0.109

#### Refinement

*R*[*F* ^2^ > 2σ(*F* ^2^)] = 0.058
*wR*(*F* ^2^) = 0.108
*S* = 1.04
3124 reflections
198 parameters
34 restraints
H atoms treated by a mixture of independent and constrained refinement
Δρ_max_ = 0.47 e Å^−3^
Δρ_min_ = −0.47 e Å^−3^

### 

Data collection: *CrystalClear* (Rigaku, 2005 ▶); cell refinement: *CrystalClear*; data reduction: *CrystalClear*; program(s) used to solve structure: *SHELXTL* (Sheldrick, 2008 ▶); program(s) used to refine structure: *SHELXTL*; molecular graphics: *SHELXTL*; software used to prepare material for publication: *SHELXTL*.

## Supplementary Material

Crystal structure: contains datablocks I, global. DOI: 10.1107/S1600536810017204/nk2032sup1.cif

Structure factors: contains datablocks I. DOI: 10.1107/S1600536810017204/nk2032Isup2.hkl

Additional supplementary materials:  crystallographic information; 3D view; checkCIF report

## Figures and Tables

**Table 1:** Hydrogen-bond geometry (Å, °)

*D*—H⋯*A*	*D*—H	H⋯*A*	*D*⋯*A*	*D*—H⋯*A*
N2—H2⋯Br2	0.91 (7)	2.23 (7)	3.141 (5)	176 (6)

**Fig. 1.**
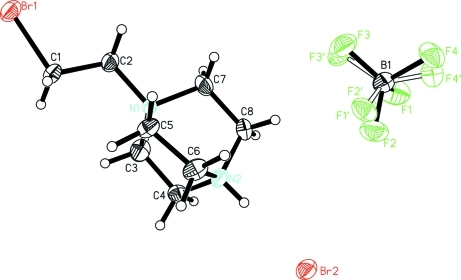
The asymmetric unit of the title compound, showing the atomic numbering scheme. Displacement ellipsoids are drawn at the 30% probability level and the minor disorder component is omitted.

## supplementary crystallographic information

### Comment

The variable-temperature dielectric response, especially in relatively high frequency range, is very useful for searching phase transitions, in which there is a dielectric anomaly at the transition temperature. Unluckily, the title compound has no dielectric disuniform from 93 K to 453 K( m.p. > 473 K ) and report here.

In this report we have established unambiguously the structure at 293 K of the title compound in the solid state by X-ray diffraction analysis. As shown in Fig. 1. Single crystal X-ray analysis reveals that there are weak hydrogen bonds between 1-(2-bromoethyl)-1,4-diazabicyclo[2.2.2]octane-1,4-diium cation and bromide anion.

For the crystal structures and properties of related compounds, see: Chen *et al.* (2009); Fu *et al.* (2009); Zhao *et al.* (2008).

### Experimental

1,4-Diazabicyclo [2.2.2]octane (5.6 g, 0.05 mol) was dissolved in 20 ml of chloroform. To this solution 0.048 mol of 1,2-dibromoethane was added at once and the mixture was refluxed for 8 hours. On standing for 16 hours at room temperature, colorless  crystals were obtained in large quantity. The crude product was collected and dissolved in 20 ml methanol, and 10ml HBF_4_ (1 mol/L) in methanol was added slowly with stirring, while white precipitate formed at once.The suspension was filtered, and dissolved in H_2_O, After a few weeks, colorless crystals were obtained by slow evaporation.

### Refinement

Positional parameters of all the H atoms except for H2 were calculated geometrically and the H atoms were set to ride on the C atoms to which they are bonded, with Uiso(H) = 1.2Ueq(C). The H2 on the N2 was freely refined.  The BF4- anion was refined using a two-part disorder model with a major:minor occupancy ratio of 84:16%.  Distance similarity and mild displacement parameter restraints were applied to the minor component.

### Crystal data

**Table:**

C_8_H_17_BrN_2_^2+^·BF_4_^−^·Br^−^	*F*(000) = 1520
*M**_r_* = 387.87	*D*_x_ = 1.886 Mg m^−^^3^
Orthorhombic, *P**b**c**a*	Mo *K*α radiation, λ = 0.71073 Å
Hall symbol: -P 2ac 2ab	Cell parameters from 5732 reflections
*a* = 11.972 (2) Å	θ = 3.7–27.5°
*b* = 10.782 (2) Å	µ = 5.96 mm^−^^1^
*c* = 21.165 (4) Å	*T* = 293 K
*V* = 2732.0 (10) Å^3^	Prism, colourless
*Z* = 8	0.3 × 0.25 × 0.2 mm

### Data collection

**Table:**

Rigaku Mercury2 diffractometer	3124 independent reflections
Radiation source: fine-focus sealed tube	1881 reflections with *I* > 2σ(*I*)
graphite	*R*_int_ = 0.109
CCD_Profile_fitting scans	θ_max_ = 27.5°, θ_min_ = 3.2°
Absorption correction: multi-scan (*CrystalClear*; Rigaku, 2005)	*h* = −15→15
*T*_min_ = 0.191, *T*_max_ = 0.303	*k* = −14→14
26313 measured reflections	*l* = −27→27

### Refinement

**Table:**

Refinement on *F*^2^	Primary atom site location: structure-invariant direct methods
Least-squares matrix: full	Secondary atom site location: difference Fourier map
*R*[*F*^2^ > 2σ(*F*^2^)] = 0.058	Hydrogen site location: inferred from neighbouring sites
*wR*(*F*^2^) = 0.108	H atoms treated by a mixture of independent and constrained refinement
*S* = 1.04	*w* = 1/[σ^2^(*F*_o_^2^) + (0.0229*P*)^2^ + 7.8245*P*] where *P* = (*F*_o_^2^ + 2*F*_c_^2^)/3
3124 reflections	(Δ/σ)_max_ < 0.001
198 parameters	Δρ_max_ = 0.47 e Å^−^^3^
34 restraints	Δρ_min_ = −0.47 e Å^−^^3^

### Special details

**Table:**

Geometry. All esds (except the esd in the dihedral angle between two l.s. planes) are estimated using the full covariance matrix. The cell esds are taken into account individually in the estimation of esds in distances, angles and torsion angles; correlations between esds in cell parameters are only used when they are defined by crystal symmetry. An approximate (isotropic) treatment of cell esds is used for estimating esds involving l.s. planes.
Refinement. Refinement of F^2^ against ALL reflections. The weighted R-factor wR and goodness of fit S are based on F^2^, conventional R-factors R are based on F, with F set to zero for negative F^2^. The threshold expression of F^2^ > 2sigma(F^2^) is used only for calculating R-factors(gt) etc. and is not relevant to the choice of reflections for refinement. R-factors based on F^2^ are statistically about twice as large as those based on F, and R- factors based on ALL data will be even larger.

### Fractional atomic coordinates and isotropic or equivalent isotropic displacement parameters (Å^2^)

**Table:**

	*x*	*y*	*z*	*U*_iso_*/*U*_eq_	Occ. (<1)
Br1	0.71717 (5)	0.02659 (6)	0.01789 (3)	0.0522 (2)	
Br2	1.27841 (5)	0.46079 (6)	0.27319 (3)	0.0510 (2)	
C1	0.7924 (4)	0.1425 (6)	0.0745 (3)	0.0471 (15)	
H1A	0.7844	0.2268	0.0592	0.057*	
H1B	0.7609	0.1375	0.1167	0.057*	
C2	0.9138 (4)	0.1047 (5)	0.0752 (3)	0.0411 (14)	
H2A	0.9423	0.1082	0.0324	0.049*	
H2B	0.9189	0.0193	0.0893	0.049*	
C3	0.9635 (5)	0.1612 (6)	0.1856 (2)	0.0523 (17)	
H3A	0.9787	0.0751	0.1957	0.063*	
H3B	0.8853	0.1774	0.1942	0.063*	
C4	1.0354 (5)	0.2443 (6)	0.2262 (2)	0.0464 (15)	
H4A	0.9891	0.3048	0.2478	0.056*	
H4B	1.0741	0.1953	0.2578	0.056*	
C5	0.9738 (5)	0.3202 (5)	0.1038 (3)	0.0402 (14)	
H5A	0.9839	0.3358	0.0590	0.048*	
H5B	0.8991	0.3465	0.1154	0.048*	
C6	1.0584 (5)	0.3924 (5)	0.1409 (3)	0.0466 (15)	
H6A	1.1116	0.4305	0.1124	0.056*	
H6B	1.0213	0.4578	0.1644	0.056*	
C7	1.1075 (4)	0.1500 (6)	0.1040 (3)	0.0512 (16)	
H7A	1.1266	0.1717	0.0609	0.061*	
H7B	1.1174	0.0612	0.1090	0.061*	
C8	1.1838 (4)	0.2187 (6)	0.1496 (3)	0.0491 (16)	
H8A	1.2188	0.1603	0.1783	0.059*	
H8B	1.2422	0.2612	0.1263	0.059*	
N1	0.9874 (3)	0.1842 (4)	0.11705 (17)	0.0266 (10)	
N2	1.1176 (4)	0.3085 (5)	0.1852 (2)	0.0397 (12)	
H2	1.165 (6)	0.349 (6)	0.212 (3)	0.09 (2)*	
B1	0.4770 (8)	0.2507 (9)	0.0910 (4)	0.0421 (19)	0.837 (14)
F1	0.5239 (8)	0.1669 (6)	0.1311 (3)	0.086 (2)	0.837 (14)
F2	0.4322 (7)	0.3475 (6)	0.1246 (4)	0.074 (2)	0.837 (14)
F3	0.3930 (7)	0.1935 (9)	0.0574 (5)	0.090 (3)	0.837 (14)
F4	0.5562 (5)	0.2945 (7)	0.0483 (3)	0.0681 (19)	0.837 (14)
B1'	0.460 (3)	0.244 (3)	0.0923 (13)	0.0421 (19)	0.163 (14)
F1'	0.454 (3)	0.186 (2)	0.1488 (12)	0.067 (7)	0.163 (14)
F2'	0.401 (3)	0.353 (3)	0.0918 (18)	0.065 (8)	0.163 (14)
F3'	0.419 (4)	0.168 (5)	0.0460 (19)	0.082 (9)	0.163 (14)
F4'	0.569 (2)	0.276 (4)	0.078 (2)	0.084 (9)	0.163 (14)

### Atomic displacement parameters (Å^2^)

**Table:**

	*U*^11^	*U*^22^	*U*^33^	*U*^12^	*U*^13^	*U*^23^
Br1	0.0483 (4)	0.0598 (4)	0.0485 (4)	−0.0138 (3)	−0.0101 (3)	−0.0049 (3)
Br2	0.0442 (4)	0.0533 (4)	0.0555 (4)	0.0022 (3)	−0.0125 (3)	−0.0125 (3)
C1	0.039 (3)	0.056 (4)	0.046 (4)	−0.010 (3)	−0.010 (3)	−0.010 (3)
C2	0.036 (3)	0.039 (3)	0.048 (4)	−0.005 (3)	−0.004 (3)	−0.006 (3)
C3	0.051 (4)	0.072 (4)	0.033 (3)	−0.020 (3)	0.002 (3)	0.015 (3)
C4	0.047 (3)	0.067 (4)	0.025 (3)	0.003 (3)	0.000 (3)	0.001 (3)
C5	0.048 (3)	0.027 (3)	0.045 (3)	0.006 (3)	−0.016 (3)	−0.003 (3)
C6	0.064 (4)	0.029 (3)	0.047 (4)	−0.006 (3)	−0.007 (3)	0.002 (3)
C7	0.034 (3)	0.052 (4)	0.067 (4)	0.009 (3)	0.008 (3)	−0.017 (3)
C8	0.028 (3)	0.062 (4)	0.057 (4)	0.001 (3)	0.002 (3)	−0.006 (3)
N1	0.026 (2)	0.026 (2)	0.028 (2)	−0.0026 (19)	0.0001 (18)	−0.0006 (18)
N2	0.036 (3)	0.049 (3)	0.034 (3)	−0.011 (2)	−0.004 (2)	−0.002 (2)
B1	0.043 (5)	0.041 (4)	0.043 (5)	−0.004 (4)	−0.009 (4)	0.000 (3)
F1	0.125 (7)	0.079 (4)	0.055 (4)	0.022 (4)	−0.005 (4)	0.019 (3)
F2	0.087 (5)	0.054 (3)	0.080 (5)	0.002 (3)	0.021 (4)	−0.022 (4)
F3	0.079 (5)	0.118 (6)	0.073 (5)	−0.021 (4)	−0.002 (4)	−0.040 (4)
F4	0.063 (3)	0.090 (4)	0.051 (4)	0.003 (3)	0.012 (3)	0.017 (3)
B1'	0.043 (5)	0.041 (4)	0.043 (5)	−0.004 (4)	−0.009 (4)	0.000 (3)
F1'	0.092 (17)	0.063 (13)	0.047 (13)	0.037 (13)	0.009 (12)	−0.001 (11)
F2'	0.064 (15)	0.048 (12)	0.082 (18)	−0.007 (12)	0.028 (14)	0.004 (14)
F3'	0.096 (18)	0.097 (17)	0.052 (15)	−0.041 (16)	0.000 (15)	−0.040 (13)
F4'	0.053 (14)	0.099 (16)	0.101 (19)	−0.009 (13)	0.001 (15)	0.015 (17)

### Geometric parameters (Å, °)

**Table:**

Br1—C1	1.952 (5)	C6—H6A	0.9700
C1—C2	1.509 (7)	C6—H6B	0.9700
C1—H1A	0.9700	C7—N1	1.510 (6)
C1—H1B	0.9700	C7—C8	1.521 (8)
C2—N1	1.515 (6)	C7—H7A	0.9700
C2—H2A	0.9700	C7—H7B	0.9700
C2—H2B	0.9700	C8—N2	1.461 (7)
C3—N1	1.499 (6)	C8—H8A	0.9700
C3—C4	1.511 (7)	C8—H8B	0.9700
C3—H3A	0.9700	N2—H2	0.91 (7)
C3—H3B	0.9700	B1—F1	1.361 (9)
C4—N2	1.483 (7)	B1—F2	1.371 (9)
C4—H4A	0.9700	B1—F3	1.377 (9)
C4—H4B	0.9700	B1—F4	1.393 (9)
C5—C6	1.500 (7)	B1'—F1'	1.354 (19)
C5—N1	1.502 (6)	B1'—F2'	1.364 (19)
C5—H5A	0.9700	B1'—F3'	1.370 (19)
C5—H5B	0.9700	B1'—F4'	1.388 (19)
C6—N2	1.484 (7)		
			
C2—C1—Br1	106.1 (4)	N1—C7—C8	109.7 (4)
C2—C1—H1A	110.5	N1—C7—H7A	109.7
Br1—C1—H1A	110.5	C8—C7—H7A	109.7
C2—C1—H1B	110.5	N1—C7—H7B	109.7
Br1—C1—H1B	110.5	C8—C7—H7B	109.7
H1A—C1—H1B	108.7	H7A—C7—H7B	108.2
C1—C2—N1	114.4 (4)	N2—C8—C7	108.9 (4)
C1—C2—H2A	108.7	N2—C8—H8A	109.9
N1—C2—H2A	108.7	C7—C8—H8A	109.9
C1—C2—H2B	108.7	N2—C8—H8B	109.9
N1—C2—H2B	108.7	C7—C8—H8B	109.9
H2A—C2—H2B	107.6	H8A—C8—H8B	108.3
N1—C3—C4	110.1 (4)	C3—N1—C5	108.8 (4)
N1—C3—H3A	109.6	C3—N1—C7	108.5 (4)
C4—C3—H3A	109.6	C5—N1—C7	107.9 (4)
N1—C3—H3B	109.6	C3—N1—C2	111.2 (4)
C4—C3—H3B	109.6	C5—N1—C2	112.3 (4)
H3A—C3—H3B	108.2	C7—N1—C2	108.0 (4)
N2—C4—C3	108.8 (4)	C8—N2—C4	110.7 (5)
N2—C4—H4A	109.9	C8—N2—C6	109.7 (5)
C3—C4—H4A	109.9	C4—N2—C6	109.7 (4)
N2—C4—H4B	109.9	C8—N2—H2	108 (4)
C3—C4—H4B	109.9	C4—N2—H2	106 (4)
H4A—C4—H4B	108.3	C6—N2—H2	113 (4)
C6—C5—N1	109.6 (4)	F1—B1—F2	110.1 (7)
C6—C5—H5A	109.8	F1—B1—F3	109.0 (7)
N1—C5—H5A	109.8	F2—B1—F3	108.8 (7)
C6—C5—H5B	109.8	F1—B1—F4	110.4 (7)
N1—C5—H5B	109.8	F2—B1—F4	110.1 (7)
H5A—C5—H5B	108.2	F3—B1—F4	108.3 (7)
N2—C6—C5	109.7 (4)	F1'—B1'—F2'	112 (2)
N2—C6—H6A	109.7	F1'—B1'—F3'	109 (2)
C5—C6—H6A	109.7	F2'—B1'—F3'	109 (2)
N2—C6—H6B	109.7	F1'—B1'—F4'	111 (2)
C5—C6—H6B	109.7	F2'—B1'—F4'	106 (2)
H6A—C6—H6B	108.2	F3'—B1'—F4'	109 (2)
			
Br1—C1—C2—N1	−179.9 (4)	C8—C7—N1—C5	63.8 (6)
N1—C3—C4—N2	−8.1 (7)	C8—C7—N1—C2	−174.6 (5)
N1—C5—C6—N2	−7.7 (6)	C1—C2—N1—C3	70.8 (6)
N1—C7—C8—N2	−7.9 (7)	C1—C2—N1—C5	−51.3 (6)
C4—C3—N1—C5	−54.1 (6)	C1—C2—N1—C7	−170.2 (5)
C4—C3—N1—C7	63.1 (6)	C7—C8—N2—C4	65.3 (6)
C4—C3—N1—C2	−178.3 (5)	C7—C8—N2—C6	−55.8 (6)
C6—C5—N1—C3	63.3 (6)	C3—C4—N2—C8	−56.3 (6)
C6—C5—N1—C7	−54.3 (6)	C3—C4—N2—C6	64.9 (6)
C6—C5—N1—C2	−173.2 (4)	C5—C6—N2—C8	65.9 (6)
C8—C7—N1—C3	−53.9 (6)	C5—C6—N2—C4	−55.9 (6)

### Hydrogen-bond geometry (Å, °)

**Table:**

*D*—H···*A*	*D*—H	H···*A*	*D*···*A*	*D*—H···*A*
N2—H2···Br2	0.91 (7)	2.23 (7)	3.141 (5)	176 (6)
